# Avalanche criticality during ferroelectric/ferroelastic switching

**DOI:** 10.1038/s41467-020-20477-6

**Published:** 2021-01-12

**Authors:** Blai Casals, Guillaume F. Nataf, Ekhard K. H. Salje

**Affiliations:** 1 Department of Earth Sciences, Cambridge University, Cambridge, UK; 2 Department of Materials Science, Cambridge University, Cambridge, UK

**Keywords:** Ferroelectrics and multiferroics, Phase transitions and critical phenomena

## Abstract

Field induced domain wall displacements define ferroelectric/ferroelastic hysteresis loops, which are at the core of piezoelectric, magnetoelectric and memristive devices. These collective displacements are scale invariant jumps with avalanche characteristics. Here, we analyse the spatial distribution of avalanches in ferroelectrics with different domain and transformation patterns: Pb(Mg_1/3_Nb_2/3_)O_3_–PbTiO_3_ contains complex domains with needles and junction patterns, while BaTiO_3_ has parallel straight domains. Nevertheless, their avalanche characteristics are indistinguishable. The energies, areas and perimeters of the switched regions are power law distributed with exponents close to predicted mean field values. At the coercive field, the area exponent decreases, while the fractal dimension increases. This fine structure of the switching process has not been detected before and suggests that switching occurs via criticality at the coercive field with fundamentally different switching geometries at and near this critical point. We conjecture that the domain switching process in ferroelectrics is universal at the coercive field.

## Abstract (web summary)

While classical approaches rely on the study of individual ferroelectric domain wall movement on long time scales, the authors consider collective movements of domain walls during short time scales, characterized by discrete jumps, as indicators of avalanches on a broad range of scales.

## Introduction

In ferroelectric materials, domain walls separate regions with different polarization directions. Their dynamical behaviour is subject to intense fundamental and applied research as it is at the core of ferroelectric switching^1–4^, which is the key design parameter, e.g., in piezoelectric^5,6^, magnetoelectric^7–9^ and memristive^10–12^ devices, as well as in devices operating above GHz frequencies^13^. Furthermore, the recent research on two dimensional functionalities offered by ferroelectric domain walls^14–17^ will find further applications once strategies to dynamically deploy the functionalities, through the motion of domain walls, have been found.

Therefore, understanding the response of domain walls to electric fields is particularly appealing. With this aim, two distinct approaches have been developed. The first one relies on the study of individual domain wall movement on long-time scales. It describes the nucleation and growth of domains and their interaction with defects, interfaces and existing domain walls^18–20^. The second one aims to understand collective movements during short-time scales^21,22^. It considers discrete impulsive jumps, or ‘jerks’, occurring during the motion of domain walls, as indicators of avalanches on a broad range of scales. In such ferroelectric avalanches, any switching event is likely to trigger subsequent switching, and anisotropic long-range interactions between local events are essential, in contrast with classical microscopic ferroelectric models^23,24^. This approach is particularly relevant for the development of neuromorphic computing architectures^25^ where maximal computational performances are achieved through scale-invariant avalanches that develop at a critical point^26,27^, similar to neuronal avalanches observed in the brain^28^. The key question is whether this switching dynamics is a universal process in ferroelectrics with little or no influence from symmetry or structural features of domain walls.

Mean-field (MF) theory applied to ferroelectrics predicts the power-law distribution of jerks^29^, which is one of the signatures of scale-free processes, and atomistic simulations show that avalanches are induced by kinks and domain walls junctions^30–32^, as well as defects acting as pinning centres^33^. Ultraslow processes have been modelled^34^ and reveal that many of the relevant ferroelectric parameters, e.g., the size of the switching events, follow power-law dependences under switching conditions. In order to confirm the origin of the avalanches, optical visualization is required and has been successfully applied during the phase transition of martensitic materials^35–37^ and during switching in magnetic thin films^38^, but not in ferroelectric materials where avalanches are usually studied through indirect methods, e.g., acoustic emission^22^ or displacement current measurements^39,40^. Previous to these observations, avalanche studies already revealed the stepwise behaviour of domain wall movements under electric fields but did not lead to a quantitative analysis of avalanche characteristics^41–43^.

In this work, we combine optical microscopy and statistical analyses to characterize the motion of ferroelastic domain wall during ferroelectric switching in two ferroelectric materials: tetragonal BaTiO_3_ (BTO), for which avalanches have already been studied with acoustic emission^22^, and monoclinic Pb(Mg_1/3_Nb_2/3_)O_3_–PbTiO_3_ (PMN-PT) close to the morphotropic boundary^44^. They show very different ferroelastic domain and transformation patterns: BTO is dominated by parallel straight charged domain walls (which we have already characterized in a previous work^45^), while in PMN-PT straight walls intersections lead to complex domain walls, which are often bent, forming needle and junction patterns whose switching differ greatly depending on the starting domain structure^46^. Our optical observations bring the experimental evidence that avalanches are triggered at kinks and domain wall junctions. Furthermore, our ability to image directly the avalanches gives us the possibility to explore the spatiotemporal changes of the ferroelectric domain pattern to identify switched regions. We find that the areas of the switched regions, their energies and perimeters are power law distributed with power-law exponents close to the predicted MF values. We also show that during ferroelectric switching, the fractal dimension of the switched regions increases and reaches a maximum at the coercive field. This fine structure of the switching process has not been detected before. Our results suggest that the change of the fractal dimension is the key observable to understand ferroelectric domain motion under electric field and that switching occurs via criticality at the coercive field with fundamentally different switching geometries at and near this critical point. This behaviour is seen in PMN-PT and BTO so that we conjecture that the overall domain switching process in ferroelectrics may be universal and show little dependence on the details of the actual domain patterns.

## Results

**Fig. 1:**
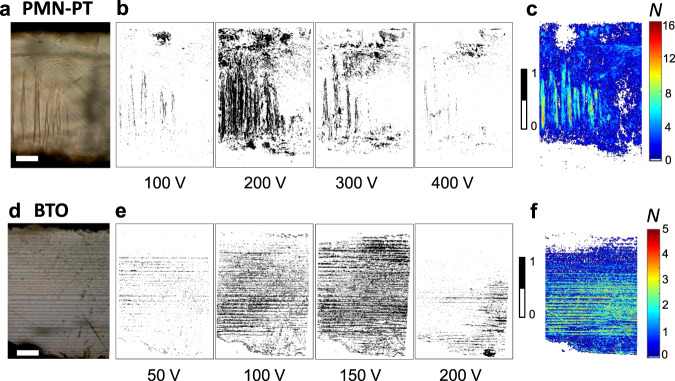
Localization of avalanches for PMN-PT and BTO. Optical image of the initial state of the sample for **a** PMN-PT and **d** BTO. The scale bar indicates 200 µm. Switched regions for **b** PMN-PT and **e** BTO, at representative applied voltages. The black regions (assigned to 1) are regions that switched between *t* and *t* + δ*t*, with δ*t* = 1/30 s, while the white regions (assigned to 0) remained unchanged. Accumulated activity maps after a full hysteresis loop for **c** PMN-PT and **f** BTO. The colour scale indicates the number of times each pixel (~1 µm^2^ in size) has been changed.

Figure 1a, d shows two domain patterns of PMN-PT and BTO, visualized with an optical microscope in transmission mode, as representative examples. The regions switching during the application of a positive voltage, i.e. the ferroelectric avalanches, are shown in Fig. 1b, e. For low voltages (<200 V for PMN-PT and <120 V for BTO), switched regions, defined as topological connected entities where pixels intensity change, form patches of small areas, around the domain walls. On approaching the coercive field (250 V for PMN-PT and 170 V for BTO), the number of switched regions increases and their area distribution becomes broader. At even higher fields (~350 V for PMN-PT and ~250 V for BTO), virtually no switching occurs and the number of switched regions decreases accordingly. After a full hysteresis loop, the accumulation maps (Fig. 1c, f) show the location of the avalanches through the number of times each pixel has been changed, i.e., the activity. These maps show that almost the entire field of view has been switched and that most changes occur around domain walls. The low activity far from the coercive field indicates that most of the intensity variations induced by the linear electro-optic effect are too small to be detected and remain within noise level.

The accumulated activity is higher in PMN-PT with its high concentration of bent domain walls, junctions, kinks and intersections (Supplementary Note 1), confirming that avalanches are triggered at kinks and domain wall junctions^30–32^. These patterns differ greatly from the domain patterns in BTO (Fig. 1d) where domain walls are much straighter and form few intersections. Nevertheless, small domain patches also form in BTO in front of advancing domain boundaries, which are topologically very similar to the domain patches in PMN-PT (an example for a single domain in BTO is shown in the Supplementary Note 5).

**Fig. 2:**
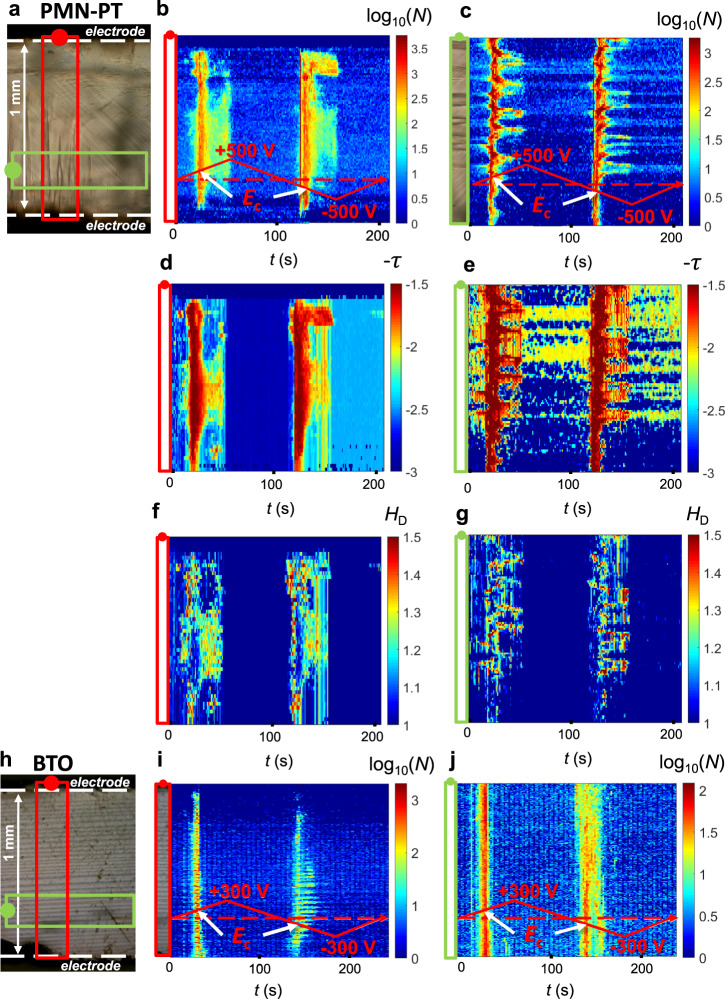
Spatiotemporal avalanche maps. Spatiotemporal avalanche maps for **a**–**g** PMN-PT and **h**–**j** BTO samples. The red and green rectangles in the optical images **a** and **h** are analysed further in **b**, **d**, **f**, **i** (red) and **c**, **e**, **g**, **j** (green). Accumulated avalanche activities integrated over space *N* for **b**, **c** PMN-PT and **i**, **j** BTO. **d**, **e** The avalanche exponent *τ* and **f**, **g** the Hausdorff dimension *H*_D_ are shown for PMN-PT. Similar graphs for BTO are shown in the Supplementary Note 4.

In Fig. 2, spatiotemporal avalanche maps of PMN-PT (Fig. 2a–g) and BTO (Fig. 2h–j) are shown. In Fig. 2b, c and Fig. 2i, j we observe that most avalanches occur at the coercive field (250 V for PMN-PT and 170 V for BTO) and that they are located in the vicinity of domain walls. We established a data set of the areas *A* of all switched regions and the corresponding energies as the square of the areas (*E* = *A*^2^). We also determine the perimeter *P* of the switched regions. Scaling *P* as a function of *A* yields the Hausdorff dimension H_D_ with $P\sim A^{H_{\mathrm{D}}/2}$ (ref. ^47^, Supplementary Notes 2 and 3), shown in Fig. 2f, g. We calculate the area exponent *τ* assuming a power-law distribution of the area with the probability distribution per area interval PDF(*A*) ~ *A*^−*τ*^, as shown in Fig. 2d, e. At the coercive field we find *τ* = 1.7 while the overall exponent is above 2. By comparing Fig. 2d, e and Fig. 2f, g, the anticorrelation between *τ* and *H*_D_ is seen when the number of switched regions is high. In the creep regime, at high voltages, we observe few small changes of the domain patterns. The same behaviour, including the anticorrelation between *τ* and *H*_D_, holds independently of the domain pattern in any of the analysed regions. In particular, in PMN-PT, the upper part of the sample—dominated by straight walls—and the lower part of the sample—dominated by needle and junction patterns—behave similarly (Fig. 2b, d, f).

**Fig. 3:**
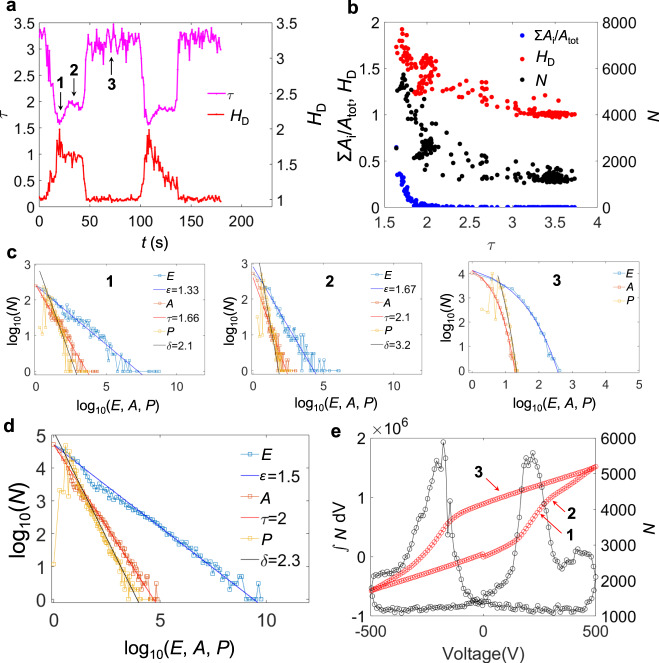
Scale-invariant avalanches in PMN-PT. **a** *H*_D_ and *τ* as a function of time. **b** *H*_D_, Σ(*A*_i_)/*A*_tot_ (proportion of switched regions with respect to the total area) and *N* (avalanche activity, defined as the number of switched regions between *t* and *t* + δ*t*) as a function of *τ*. **c** *E* (energy), *A* (area) and *P* (perimeter) distribution at the time steps marked as 1, 2 and 3 by arrows in **a**. **d** *E*, *A* and *P* distribution from all the time steps. **e** *N* as a function of the applied voltage (right axis) and its integration over the voltage applied (left axis).

**Fig. 4:**
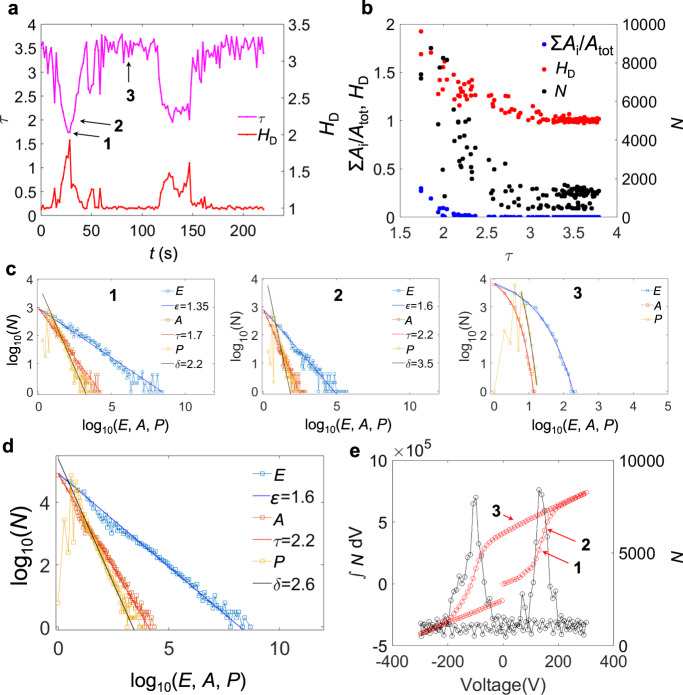
Scale-invariant avalanches in BTO. **a** *H*_D_ and *τ* as a function of time. **b** *H*_D_, Σ(*A*_i_)/*A*_tot_ (proportion of switched regions with respect to the total area) and *N* (avalanche activity, defined as the number of switched regions between t and *t* + δ*t*) as a function of *τ*. **c** *E* (energy), *A* (area) and *P* (perimeter) distribution at the time steps marked as 1, 2 and 3 by arrows in **a**. **d** *E*, *A* and *P* distribution from all the time steps. **e** *N* as a function of the applied voltage (right axis) and its integration over the voltage applied (left axis).

In Figs. 3 and 4 we plot the log–log dependences of the area, energy and perimeter for both materials together with their Hausdorff dimensions, as integrated over the full field of view. The anticorrelation between the area exponent *τ* and the Hausdorff dimension *H*_D_ becomes obvious in Figs. 3a and 4a. The distribution of area, energy and perimeter at selected representative points (numbers 1, 2 and 3) in the different regimes are shown in Figs. 3c and 4c. At the coercive field, the switching operates with the lowest *τ* (regime 1), while *τ* increases in the vicinity, but away from the coercive field value (regime 2). The *τ*-values in the creep regime (regime 3), where the jerks are uncorrelated and do not represent power laws, are approximated by the asymptotic behaviour of the slope near the upper value of the areas (respectively energies or perimeters) in the PDF curves. These values have no direct physical meaning besides showing that in this regime no significant switching occurs. The Hausdorff dimension in this regime is *H*_D_ = 1.

The anticorrelations are quantitatively shown in Figs. 3b and 4b. At the minima of *τ* we find the maxima of *H*_D_. The minimum value of *τ* is 1.7 (energy exponent *ε* = 1.3) in both materials, the corresponding Hausdorff dimension is ~1.8. The changes of the domain patterns are highly fractal and contain a multitude of large regions with rough surfaces, which contribute greatly to the increase of the number of avalanches at minima in *τ* (Figs. 3e and 4e). With increasing *τ*-value we find that *H*_D_ decreases until the switching interval has passed and *H*_D_ approaches unity (Figs. 3a and 4a). Comparing these fine structures of the switching process with the total switching behaviour as seen in Figs. 3d and 4d, the value of *τ* ∼ 2−2.2 is obtained, in accordance with results of previous acoustic emission measurements in BTO^22^. The closeness of this exponent to that of regime 2 is understood by the larger duration of regime 2 compared with regime 1 and the high number of switching events over a broader interval (Figs. 3e and 4e). The field dependence of this activity (Figs. 3e and 4e) reproduces the ferroelectric hysteresis very well, indicating the predominant role of ferroelastic domain walls in mixed ferroelectric/ferroelastic systems.

## Discussion

Strain fields, which are long ranging and anisotropic, induce correlations that are a key element in avalanches^22^. Such correlations had already been inferred from studies of switching dynamics in polycrystalline materials, where depolarization fields are screened by adapting local bound charges and elastic interactions dominate. In these systems, ferroelastic domain walls are highly mobile compared to 180° domain walls and their synchronized movements within grains is shown to reproduce well the ferroelectric hysteresis loop^48–50^. Even in uniaxial ferroelectrics (e.g. LiNbO_3_ and LiTaO_3_), where avalanche behaviour related to the movement of non-ferroelastic 180° domain walls have been observed (but not quantified)^51,52^, it is possible that secondary local strain^53^ accompanying the movement of domain walls plays a role. In fact, in magnetic systems, it has been shown that even magnetic Barkhausen noise depends explicitly and strongly on strain coupling^54,55^.

We note that our observations for ferroelectrics are also consistent with results obtained in other critical systems, e.g., during the failure of materials where the energy exponent *ε* decreases before failure^56–58^, sometimes down to the MF value *ε* = 1.3, when the system reaches the critical point^58^ while the rate of energy released diverges^59–61^.

We have shown that ferroelectric/ferroelastic switching progresses via avalanches in two typical ferroelectric materials. We are able to correlate the visible domain switching with predictions of avalanche theory and show the close agreement with the results of previous measurements of acoustic emission in BTO^22^. The closeness of the avalanche characteristics with those predicted by MF theory^62^ is remarkable. Surprising and somewhat counterintuitive is the observation that fine structures appear during the switching process. The presumed critical point of the switching process is the coercive field. At the coercive field, the area and energy exponents correspond to an unrelaxed MF value (*ε* = 1.3, *τ* = 1.7), while the fractality is maximum with *H*_D_ = 1.8. In the creep regime, far away from the main switching, the areas are exponentially distributed which indicates uncorrelated noise and *H*_D_ = 1. In between these two regimes, the switching progresses with exponents near the field integrated MF model with *ε* = 1.6 and *τ* ~ 2.2. The switching activity in this regime is reduced but spreads over a longer time so that the overall switching follows *ε* = 1.6 and *τ* = 2.2. We do not observe exponent mixing as could be expected^63^ but our experimental resolution is probably insufficient for the investigation of mixing. Furthermore, the mixing proceeds not simply via two fix points with two exponents but via a continuous distribution of exponents which increase continuously from *ε* = 1.3 and *τ* = 1.7 to greater values. Simultaneously *H*_D_ decreases, which implies that the switched regions become more compact, with interfaces that are much smoother than those at the critical point. At these late stages of the switching process, smooth domain walls progress without much change to their shape. The universal framework presented here suggests operating ferroelectrics close to the coercive field, where criticality and scale-invariant avalanches develop, maximizing their properties.

## Methods

### Samples

The PMN-PT (001) sample, with composition (1 − *x*)[Pb(Mg_1/3_Nb_2/3_)O_3_]−*x*[PbTiO_3_], *x* = 0.32, is a commercial sample from Atom Optics Co., Ltd (Shanghai, China), with dimensions 5 × 1 × 0.5 mm. The BTO (111) sample is from MTI corporation (USA), with dimensions 6 × 1 × 1 mm.

### Optical microscopy and displacement current measurements

We visualized the ferroelectric domain structure with an optical microscope in transmission mode (Leica), using an objective with magnification ×20 and numerical aperture 0.4. The experimental setup is described in ref. ^45^, polarized white light is transmitted through the sample, and an analyser is used to select the polarization state before the CCD camera. The optical contrast between ferroelastic domains arises from the unique symmetric polar tensor of second rank determining the optical indicatrix of each ferroelastic domain state. In order to move domain walls, we applied an electric field along the [100]_pc_ direction for PMN-PT (1-mm-thick) and the [−110]_pc_ direction for BTO (1-mm-thick) with two silver-paint electrodes on the sides of the samples. We used a picoammeter (Keithley 6487) to measure the displacement current of the sample while cycling the applied electric field (Supplementary Note 6) to ensure that full switching loops are measured. We cycled the voltage (electric field) between 0 and ± 500 V for PMN-PT at 10 V s^−1^ and from 0 to ±300 V for BTO at 7 V s^−^^1^ with a triangular function while recording optical images at 30 frames per seconds (noted as δ*t* = 1/30 s). The results presented are obtained after more than 10 cycles to avoid ‘virgin’ effects.

### Statistical analysis of optical images

In order to identify the avalanches, we perform a pixel by pixel analysis of the optical images during switching. Jerks *J*_*ij*_ in intensity as a function of time are defined as (1):

1 $$J_{ij} = \left( {\frac{{{\mathrm{d}}B_{ij}}}{{{\mathrm{d}}t}}} \right)^2,$$

where *B*_*ij*_ is the intensity at each pixel (*i* and *j* are indices used for the spatial coordinates). The areas *A* of the switched regions are defined as topological connected entities where the values of *J*_*ij*_ are greater than a threshold. Detailed analysis shows that these regions contain an almost constant intensity (Supplementary Notes 2 and 3). We use as threshold a value that is twice the mean value of the pixel’s noise under the absence of electric field. The exact value of the threshold proved irrelevant for our observations.

For exponents, we use *τ* for areas, *ε* for energies and *δ* for perimeters. They are derived from the probability distribution function (PDF) as slopes of the log–log PDF curves and independently by the maximum likelihood method^64^ (Supplementary Note 5).

We compute the entropy of the avalanche images as $- \mathop {\sum }\nolimits^ p\;{\mathrm{log}}_2(p)$, where *p* contains the normalized histogram counts of switched pixels and we fit its evolution with the avalanche occupancy with a parabolic function, following percolation theory^65^ (Supplementary Notes 2 and 3).

## Supplementary information

Supplementary Information

## Data Availability

Optical microscopy videos showing the evolution of the domain structure during ferroelectric switching are available from the corresponding author on request.

## Code availability

The code used for the statistical analysis of optical images is available upon request.
