# Supplementary material for: Avalanche criticality during ferroelectric/ferroelastic switching
Source: Nat Commun. 2021 Jan 12;12:345. doi: 10.1038/s41467-020-20477-6 (PMC7804440; doi:10.1038/s41467-020-20477-6)
Supplement: Supplementary file 1 — Supplementary Information [file 41467_2020_20477_MOESM1_ESM.pdf]

SUPPLEMENTARY INFORMATION

Avalanche criticality during ferroelectric/ferroelastic switching

Blai Casals[1,*], Guillaume F. Nataf[2], Ekhard K. H. Salje[1]

[1] Department of Earth Sciences, Cambridge University, Cambridge UK
[2] Department of Materials Science, Cambridge University, Cambridge UK

*blaicasals@gmail.com

Supplementary Note 1: Domain motion under electric field and switched regions for $Pb(Mg_{1/3}Nb_{2/3})O_3$–$PbTiO_3$

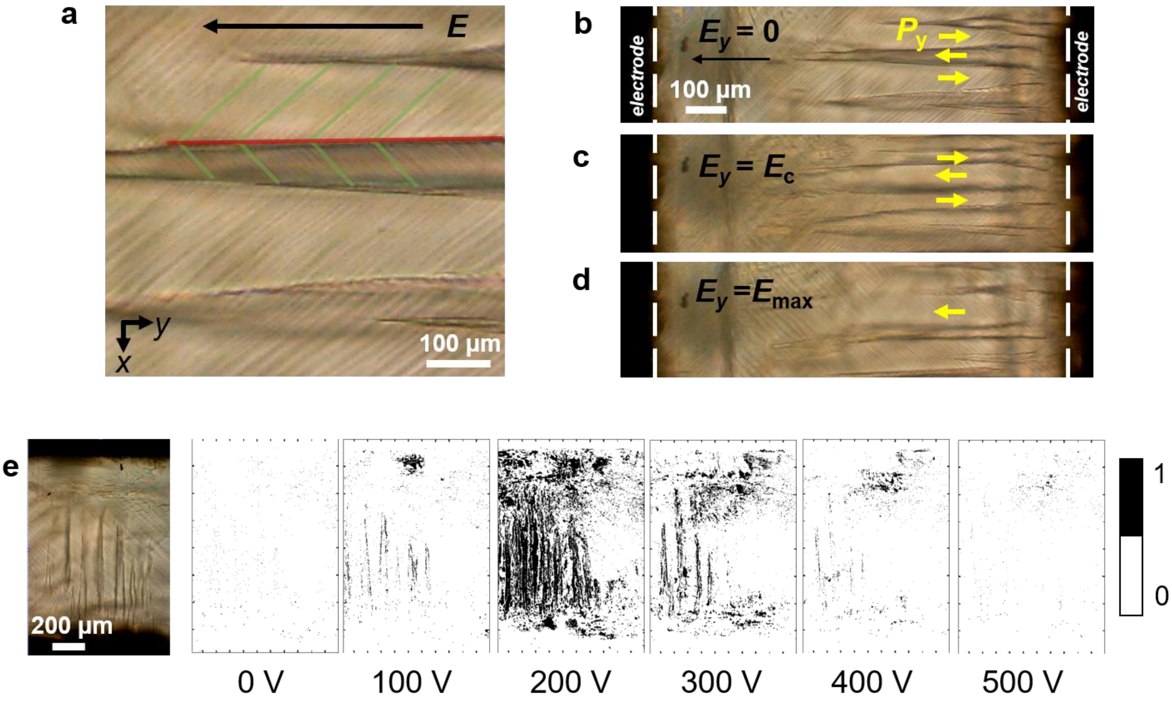

Figure S1. (a) Optical image of the initial state of the $Pb(Mg_{1/3}Nb_{2/3})O_3$-$PbTiO_3$ (001) sample, where straight domain walls with equation x = ±y (green lines) intersect (red line). Panels (b), (c) and (d) show the optical images at different applied electric field. The yellow arrows represent the direction of the polarization along the y ($[100]_{pc}$) direction. (e) Optical image of the initial state of a larger area of the sample and the corresponding switched regions at representative applied voltages. The black regions (assigned to 1) are regions that switched between t and $t+\delta t$, with δt = 1/30 s, while the white regions (assigned to 0) remained unchanged.

Supplementary Note 2: Avalanche analysis for $Pb(Mg_{1/3}Nb_{2/3})O_3–PbTiO_3$

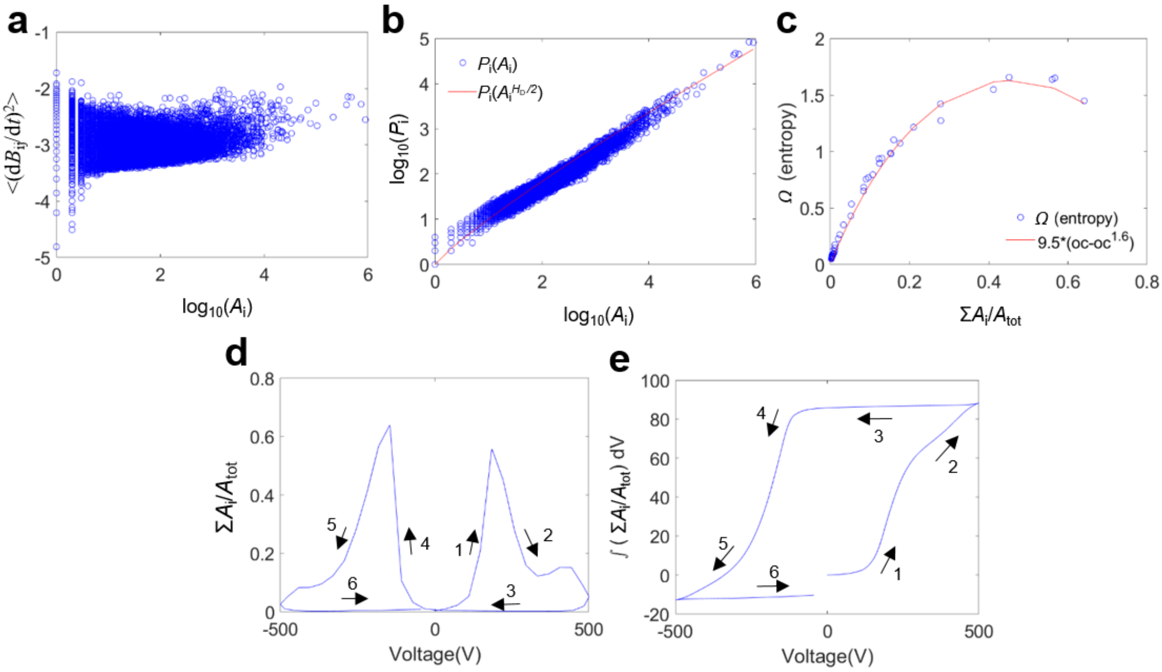

Figure S2. (a) $<(dB_{ij}/dt)^2>$ values inside each defined switched region of area A_i. (b) Correlation between perimeters P_i and areas A_i from which the Hausdorff dimension H_D is obtained. (a) and (b) show data for a full hysteresis loop. (c) Entropy of the avalanche images as a function of the area occupied by the switched regions A_i with respect to the total area A_{tot} (avalanche occupancy) for each recorded frame, i.e. every 1/30 s. The red line represents a parabolic fit. (d) Avalanche occupancy as a function of applied voltage and (e) its integration over the voltage applied. Arrows indicate the time evolution.

Supplementary Note 3: Avalanche analysis for $BaTiO_3$

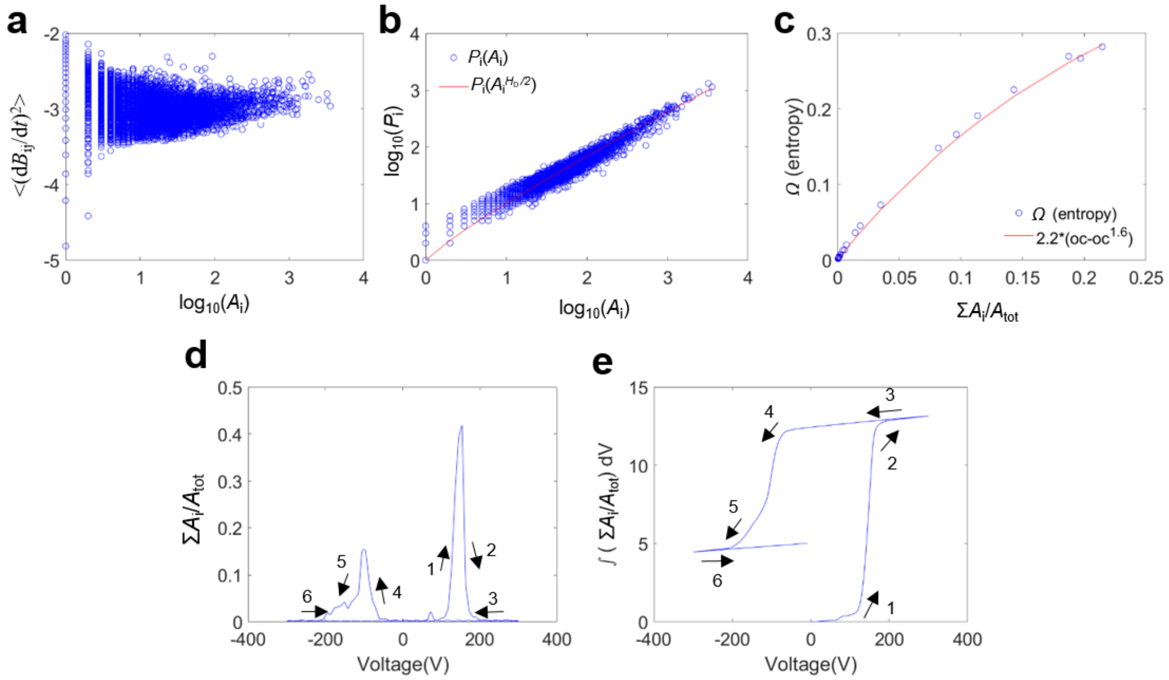

Figure S3. (a) mean values of $(dB_{ij}/dt)^2$ inside each defined switched region of area A_i. (b) Correlation between perimeters P_i and areas A_i from which the Hausdorff dimension H_D is obtained. (a) and (b) show data for a full hysteresis loop. (c) Entropy of the avalanche images as a function of the area occupied by the switched regions A_i with respect to the total area A_{tot} (avalanche occupancy) for each recorded frame, i.e. every 1/30 s. The red line represents a parabolic fit. (d) Avalanche occupancy as a function of applied voltage and (e) its integration over the voltage applied. Arrows indicate the time evolution.

Supplementary Note 4: Spatiotemporal avalanche maps for $BaTiO_3$

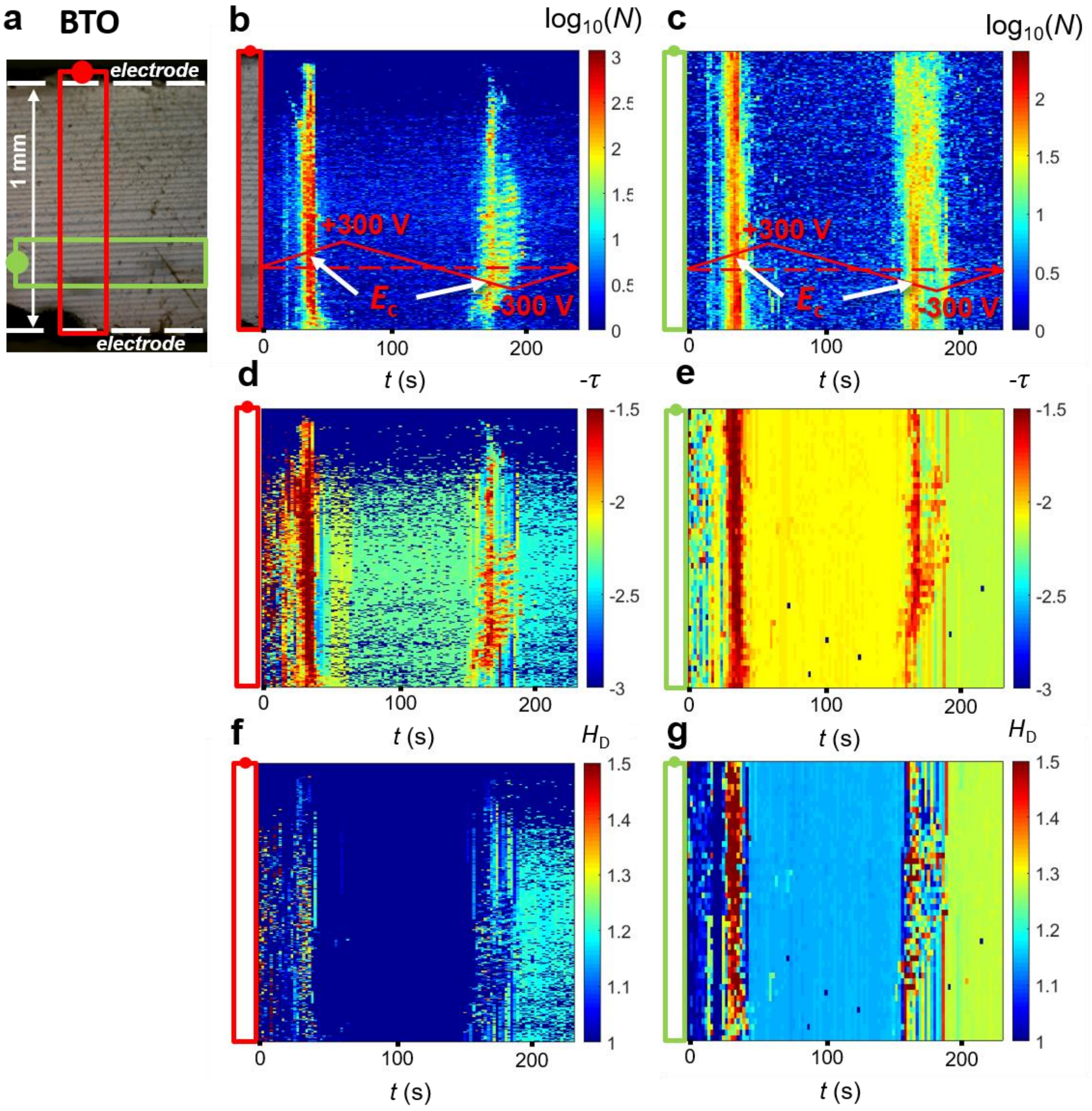

Figure S4. The red and green rectangles in the optical image a are analysed further in b, d, f (red) and c, e, g (green). (b, c) Accumulated avalanche activities integrated over space N. (d, e) Avalanche exponent τ and (f, g) Hausdorff dimension H_D.

Supplementary Note 5: Single-domain analysis for $BaTiO_3$

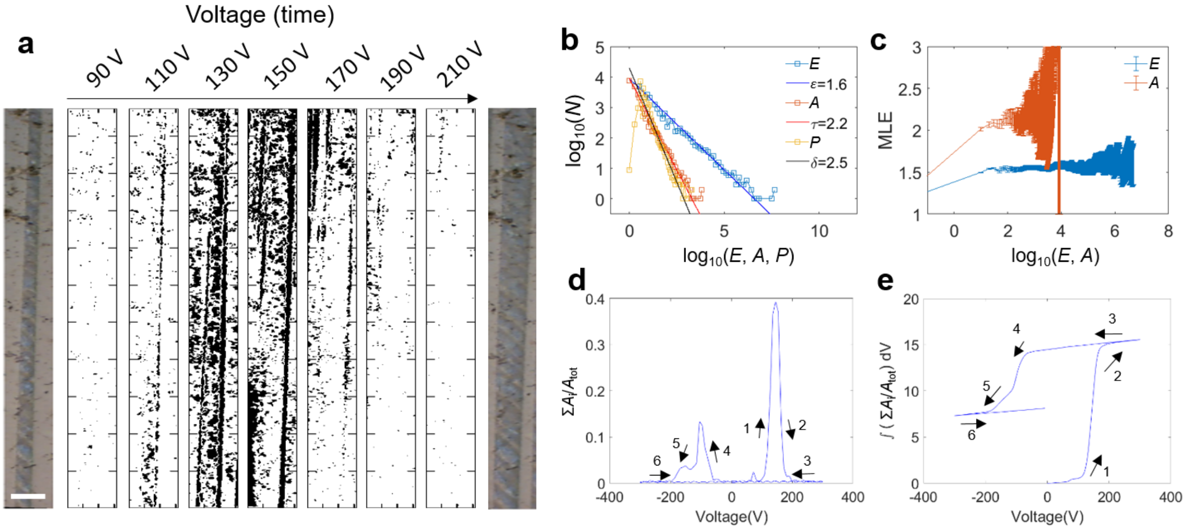

Figure S5. (a) Optical images of the initial state and final state of the $BaTiO_3$ sample after applying a positive voltage, and switched regions at representative applied voltages. The scale bar indicates 25 µm. (b) *E* (energy), *A* (area), *P* (perimeter) distribution from all the time steps obtained from a single domain. (c) Maximum likelihood exponent for E and A as a function of the lower cut-off. (d) Avalanche occupancy as a function of applied voltage and (e) its integration over the voltage applied.

Supplementary Note S6: Displacement current measured simultaneously with the optical images

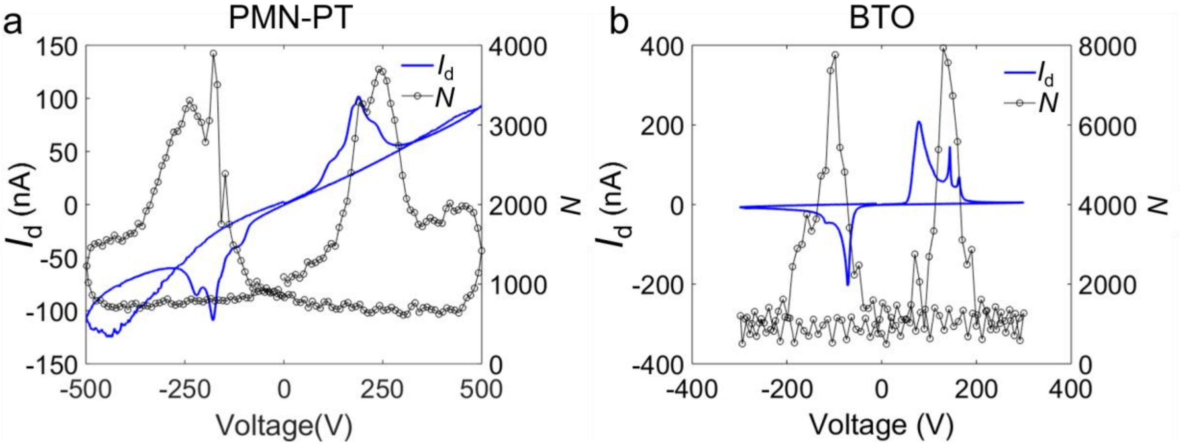

Figure S6. Displacement current I_d (blue line) measured simultaneously with the avalanche activity *N*, defined as the number of switched regions between *t* and $t+\delta t$ (black circles) for (a) $Pb(Mg_{1/3}Nb_{2/3})O_3$-$PbTiO_3$ and (b) $BaTiO_3$.
